# Serum A20 level is associated with bone mineral density in male patients with type 2 diabetes mellitus

**DOI:** 10.3389/fendo.2025.1490214

**Published:** 2025-02-26

**Authors:** Dongxu Han, Jingnan Liu, Yu Wang, Hongxia Wang, Lingdan Yuan, Wei Jin, Lige Song

**Affiliations:** ^1^ Department of Endocrinology, Shanghai Tongji Hospital, School of Medicine, Tongji University, Shanghai, China; ^2^ Institute of Osteoporosis and Metabolic Bone Diseases, School of Medicine, Tongji University, Shanghai, China

**Keywords:** A20, type 2 diabetes mellitus, bone mineral density, bone turnover markers, low bone mass

## Abstract

**Background:**

A20, also known as TNF-α-induced protein 3 (TNFAIP3), is a crucial negative regulator of inflammation and immune responses. Emerging evidence suggests that A20 is involved in the regulation of glucose metabolism and plays a significant role in bone metabolic diseases by inhibiting nuclear factor (NF)-κB activation. However, the potential relationship between serum A20 level and bone mineral density (BMD) in patients with type 2 diabetes mellitus (T2DM) has not been explored. This study aims to investigate the association between serum A20 level with BMD and bone turnover markers (BTMs) in patients with T2DM.

**Method:**

A total of 189 patients with T2DM and 183 non-diabetic individuals were included in the study based on the inclusion and exclusion criteria. Participants were categorized into normal BMD and low BMD groups. Baseline clinical histories were collected through face-to-face questionnaires. Participants underwent measurements of blood biochemistry and anthropometric, hand grip strength records and short physical performance battery (SPPB) assessment. Serum A20 level was quantified by enzyme-linked immunosorbent assay kit. Areal BMD was measured using dual-energy x-ray absorptiometry (DXA). A T-score of less than -1.0 at the lumbar spine 1-4, femoral neck and/or total hip was classified as low BMD.

**Results:**

Serum A20 level was lower in patients with T2DM compared to controls [41.30 (29.91, 61.87) vs 76.01 (54.90, 109.64) pg/mL, P<0.001]. Bivariate correlation analysis revealed that A20 level was not associated with SPPB but negatively correlated with waist-to-hip ratio (WHR). Pearson correlation analysis showed A20 level was positively correlated with lumbar spine 1-4 BMD in male diabetic patients (r=0.253, P=0.032). Multivariate regression analysis showed a positive association between serum A20 level and lumbar spine 1-4 BMD (Beta=0.047; 95% CI: 0.007-0.086; P=0.024) after multivariate adjustment. Logistic regression analysis showed that lower serum A20 level predicted low BMD in male patients with T2DM (OR: 0.22; 95% CI: 0.09-0.59; P=0.002).

**Conclusions:**

Type 2 diabetic patients exhibited lower serum A20 level compared to non-diabetic individuals. In male patients with T2DM, serum A20 level showed a significant positive correlation with lumbar spine 1-4 BMD and could serve as an independent negative predictor for low BMD.

## 1 Introduction

Type 2 diabetes mellitus (T2DM) is a widespread and increasing global health concern, characterized by hyperglycemia resulting from insulin resistance and pancreatic β-cell dysfunction (1). It has been reported that approximately 463 million adults had diabetes in 2019, of which more than 90% were T2DM (2). Osteoporosis is characterized by low bone mass and microarchitectural deterioration (3). Fragility fracture is the most severe complication of osteoporosis (4). One-third of females and one-fifth of males over 50 years old will experience fragility fractures, leading to significant economic burdens (5). Diabetes and osteoporosis often co-exist in older adults. In China, the prevalence of osteoporosis among patients with T2DM was found to be 37.8% (6). Increasing studies considered diabetic osteopathy as a complication of T2DM (7). It is widely recognized that T2DM is an independent risk factor of fragility fracture (8), which may be explained by accumulation of advanced glycation end-products (AGEs), increased adipogenesis in the bone marrow and chronic low-grade inflammation (9).

Chronic inflammation and oxidative stress act as crucial factors for the pathogenesis of both T2DM and osteoporosis. The release of the pro-inflammatory cytokine tumor necrosis factor-α (TNF-α) by obese adipose tissue plays a crucial role in contributing to the development of T2DM by impacting insulin signaling through the suppression of insulin receptor substrate 1 (IRS-1) (10). Pancreatic β cells are vulnerable to oxidative damage due to their limited antioxidant defenses, which can lead to dysfunction and apoptosis (11). Persistent hyperglycemia can stimulate the release of inflammatory cytokines, which can increase the risk of osteoporosis and bone fragility fractures through the nuclear factor-kappa B (NF-κB) pathway (12). Besides, excessive production of reactive oxygen species (ROS) induced by hyperglycemia could upregulate NF-κB signaling, leading to the polarization of macrophages towards the M1 phenotype and thus increase bone resorption (13). However, the specific mechanism of diabetic osteopathy has not yet been clarified, which leads to the limited effective therapies. Therefore, seeking novel biomarkers is of great significance for exploring potential targets for therapy. Recently, several pro-inflammatory biomarkers has been shown associated with bone homeostasis in T2DM, such as interleukin-1(IL-6), interleukin-1 (IL-1) and omentin-1 (14).

A20, also known as TNFAIP3 (Tumor Necrosis Factor Alpha-Induced Protein 3), is a zinc finger protein that functions as a critical negative regulator of inflammation and immune responses. NF-κB functions as a transcription factor that controls the activity of inflammatory signaling (15). Moreover, NF-κB serves as a detector for oxidative stress, responding to ROS activation (16). Specifically, A20 could terminate NF-κB signaling by removing ubiquitin chains from signaling intermediates such as RIP (receptor-interacting protein) (17). Increasing studies found that A20 participated in the early-onset and development of multiple inflammatory and autoimmune diseases, such as non-alcoholic fatty liver disease, rheumatoid arthritis, Behcet’s disease, etc. (18, 19). Aberrant A20 was also associated with diabetes and bone disease. A20 may regulate the gene expression of islet cells, thereby modulating insulin secretion (20). Besides, A20 could impact osteoclasts differentiation and proliferation. Enhanced A20 expression under hypoxic conditions can impede bone marrow monocytes (BMMCs) differentiation into osteoclasts (21). Mice with targeted deletion of A20 in osteoclasts exhibited severe trabecular bone loss, marked reductions in trabecular volume and osteoporosis (22).

In summary, given that chronic inflammation and oxidative stress play key roles in both T2DM and osteoporosis, it is proposed that A20 could influence the development of diabetic osteopathy by regulating inflammation. Although the bone mineral density (BMD) may underestimate fracture risk in individuals with T2DM, the evaluation of BMD through DXA remains a reliable predictor of fracture risk (23). This study aimed to investigate the correlation between serum A20 level and bone mass in patients with T2DM, which may provide novel insights for revealing the pathogenesis of diabetic osteopathy.

## 2 Materials and methods

### 2.1 Study population

**Figure 1:**
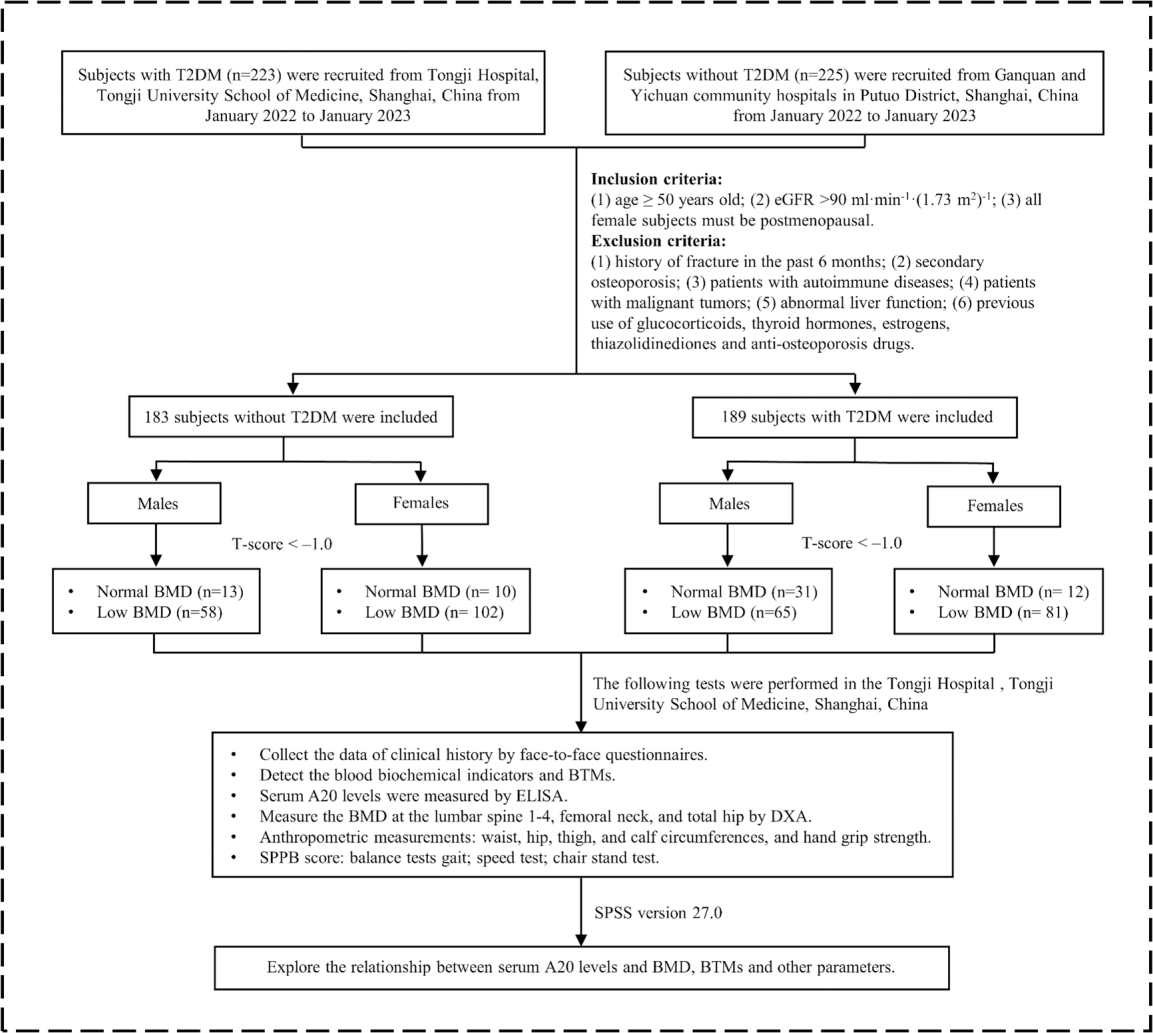
The flowchart of this study. T2DM, type 2 diabetes mellitus; eGFR, estimated glomerular filtration rate; AST, aspartate aminotransferase; ALT, alanine aminotransferase; BMD, bone mineral density; BTMs, bone turnover markers; ELISA, enzyme-linked immunosorbent assay; DXA, dual-energy x-ray absorptiometry; SPPB, short physical performance battery.

In this study, 223 patients with T2DM were recruited from Tongji Hospital, Tongji University School of Medicine, Shanghai, China from January 2022 to January 2023. At the same time, 225 patients without T2DM were recruited from Ganquan community and Yichuan community hospitals in Putuo District, Shanghai, China. T2DM was diagnosed according to the criteria by the World Health Organization (1999). Inclusion criteria were as follows (1): age ≥ 50years old (2); estimated glomerular filtration rate (eGFR) > 90 ml·min^-1^·(1.73 m^2^)^-1^ (3); all female subjects must be postmenopausal. Exclusion criteria were as follows (1): history of fracture in the past 6 months (2); secondary osteoporosis (3); patients with malignant tumors (4); patients with autoimmune diseases (5); abnormal liver function [aspartate aminotransferase (AST) > 70 U·L^-1^ or alanine aminotransferase (ALT) > 80 U·L^-1^] (6); previous use of glucocorticoids, thyroid hormones, estrogens, thiazolidinediones and all types of anti-osteoporosis drugs. The protocol of this study has been permitted by the Ethics Committee of Tongji Hospital. The trial was registered on the Chinese Clinical Trial Registry (chictr.org.cn; CTR number: ChiCTR1800018700). All patients had signed informed consent before the study started. Finally, 189 patients with T2DM and 183 patients without T2DM as the control group participated in this study (**Figure 1**).

### 2.2 Demographic data and clinical data

Demographic data was gathered by face-to-face questionnaires for all patients including age, duration of T2DM, history of diseases, current smoking, current drinking, usage of drugs, etc. Drinking status was specified as drinking three or more units of alcohol daily, with one unit equaling 8 grams of alcohol.

### 2.3 Biochemical parameters measurement

After 8-12 hours of fast, fasting blood was collected to detect hemoglobin (Hb), fasting plasma glucose (FPG), triglyceride (TG), total cholesterol (TC), low-density lipoprotein (LDL), high-density lipoprotein (HDL), serum creatinine (CREA), AST, ALT, glycated hemoglobin (HbA1c), fasting insulin (FINS), c-terminal cross-linking telopeptide of type I collagen (CTX) and procollagen type I intact N-terminal (P1NP). Hb was quantitatively detected by an automatic analyzer. The detection method was cyano-methemoglobin colorimetry. FINS level was detected by the chemiluminescence method (Roche COB8000). Electrolytes, blood lipid indexes, creatinine and liver function were measured by an automatic serum chemical analyzer. A whole blood high-performance liquid chromatography was applied to detect HbA1c. Serum P1NP and CTX were measured using electrochemiluminescence assay (Roche, Cobas601). The homeostasis model assessment for insulin resistance (HOMA-IR) was determined using the formula: [FPG (mmol/L) × FINS (μU/mL)].

### 2.4 BMD detection by DXA

Dual-energy x-ray absorptiometry (DXA, HOLOGIC Discovery; coefficient of variation <1%) was employed to detect areal BMD at specific sites (lumbar spine 1–4, femur neck and total hip). To minimize errors, each operation was performed by the same technician. The unit of BMD is grams per square centimeter (g/cm²). T scores for the BMD were calculated based on the reference young adult populations. T-score= (BMD-mean BMD of young adult of the same sex)/standard deviation of young adult BMD. A T-score of less than -1.0 at any measurement site was classified as low BMD.

### 2.5 SPPB assessment

The Short Physical Performance Battery (SPPB) was employed to evaluate the physical performance of participants (24). The SPPB score consists of the following three parts (1). Balance tests: participants performed three balance exercises: standing with their feet side-by-side, semi-tandem stand and tandem stand (2). Gait speed test: gait speed of participants was measured over a 4-meter course at their usual pace (3). Chair stand test: this component assessed lower body strength and endurance. Participants were asked to rise from a seated position five times as quickly as possible without using their arms. Each test score ranged from 0 to 4 and the total SPPB score was calculated by summing the scores from each component, yielding a range from 0 to 12, with higher scores reflecting better physical performance.

### 2.6 Grip strength and anthropometric measurements

Grip strength was assessed by an electronic grip meter and the instrument was corrected before the test. Measure the grip strength of both hands separately 3 times, with a 2-minute interval between each measurement. Record the average grip strength of both hands. The grip strength was expressed in absolute unit (kg). Weight and height were measured while wearing no shoes and light clothing. Waist circumference was measured at the level of the umbilicus, and hip circumference was measured at the widest part of the buttocks. The waist to hip ratio (WHR) was calculated to assess abdominal obesity. WHR=Waist circumference (cm)/Hip circumference (cm). Body mass index (BMI) (kg·m^-2^) =Weight (kg)/Height (m) ^2^.

### 2.7 Blood samples collection and serum A20 level qualification by ELISA

Fasting venous blood was collected by trained professionals in the morning. The samples were then centrifuged at 4°C and 3000 rpm for 5 minutes to separate the serum and stored at -80°C until analysis (no more than 6 months). Serum A20 level concentrations were measured utilizing a commercially available enzyme-linked immunosorbent assay (ELISA) kit (CUSABIO, Wuhan, China) according to the manufacturer’s protocols. The sensitivity was 5.8 pg/mL. The intra-assay and inter-assay variations were <10%.

### 2.8 Statistical analysis

Statistical analysis and visualization were performed by SPSS version 27.0 (IBM, Armonk, NY, USA) and the software GraphPad Prism version 10.0, separately. Shapiro-Wilk was applied to test the normality of the data. Mean ± SD was utilized to describe the continuous parameters in a normal distribution. Continuous parameters in a skewed distribution were shown as median (p25-p75). Categorical parameters were described as percentages and numbers. For variables in a normal distribution, t-test was employed to compare the difference between two groups. Otherwise, the Mann-Whitney U test was used for analysis. Difference analysis in categorical variables was conducted by the chi-square test. Factors with P<0.1 were considered to have potential associations with low bone mass and were included in the binary logistic regression. A20 level was normalized by ln transformation and thus was in a normal distribution. Pearson or Spearman correlation analysis was used to explore the association between serum A20 level and other clinical parameters, including BMD. Partial correlation analysis was conducted to extract the correlation coefficient after adjusting for age, sex, smoking, drinking and BMI. Univariate linear regression analysis was conducted to explore which variable was correlated with the BMD of lumbar spine 1-4. Variables with P<0.1 were considered as confounding factors and would be adjusted. Multiple linear regression analysis was employed to correct confounding factors to figure out if there was an independent correlation between serum A20 level and BMD of lumbar spine 1-4. Statistical significance was defined as a two-sided P-value <0.05.

## 3 Results

### 3.1 Serum A20 level and baseline characteristics of the studied populations

**Figure 2:**
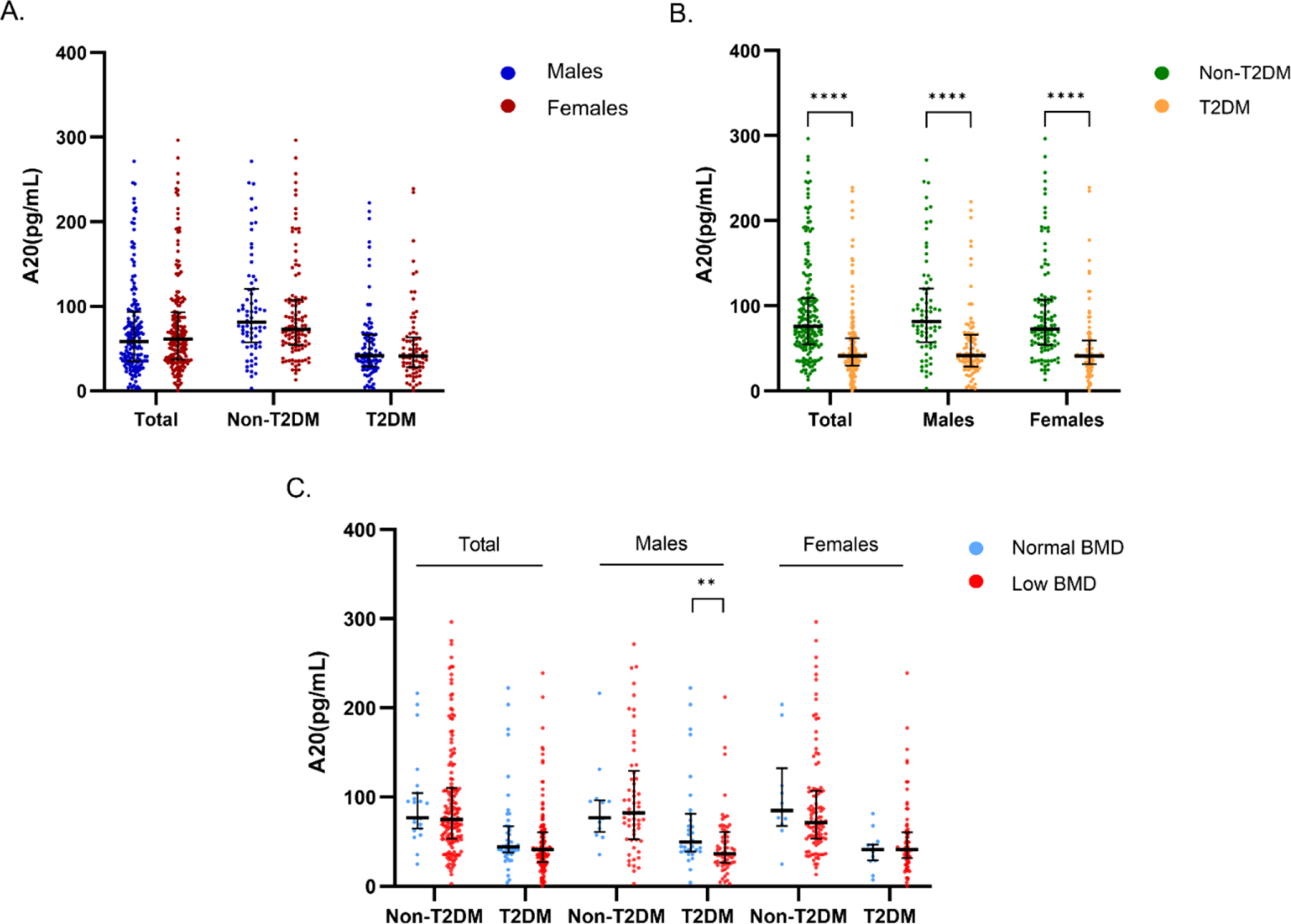
Comparison of serum A20 level grouped by gender, T2DM and bone mass. **(A)** Comparison of serum A20 level between males and females in the total, non-diabetic and diabetic populations. **(B)** Comparison of serum A20 level between non-diabetic and diabetic groups in the total population, males and females. **(C)** Comparison of serum A20 level between the normal BMD and low BMD groups categorized by sex and T2DM status. The statistical analyses were performed using Mann-Whitney U test and the significant levels were set at **P<0.01, ****P<0.0001. T2DM, type 2 diabetes mellitus; BMD, bone mineral density.

There was no significant difference in serum A20 level between males and females, regardless of diabetes status (**Figure 2A**). Compared with non-T2DM patients, serum A20 level was significantly lower in patients with T2DM in the total population [41.30 (29.91, 61.87) vs 76.01 (54.90 VS 109.64) pg/mL, P<0.001], the male individuals [41.70 (29.00, 66.25) vs 81.66 (57.65, 120.51) pg/mL, P<0.001] and the female individuals [41.30 (31.61, 59.37) vs 72.93 (54.19, 107.63) pg/mL, P<0.001] (**Figure 2B**). Further we found that in the male patients with T2DM, serum A20 level was lower in low BMD group compared with the normal BMD group [36.53 (26.56, 59.32) vs 49.47 (39.12, 77.45) pg/mL, P=0.007] (**Figure 2C**), but no significant difference was shown in serum A20 level between the normal BMD group and low BMD group in females and the total population.

In male patients with T2DM, compared to the normal BMD group, the low BMD group had lower BMI (23.86 ± 2.697 vs 25.36 ± 3.138 kg/m^2^, P=0.026), calf circumference (33.19 ± 3.496 vs 35.15 ± 2.659 cm, P=0.015) and TC (4.18 ± 1.192 vs 4.89 ± 1.546 mmol/L, P=0.029). In male patients without T2DM, compared with the normal BMD group, the low BMD group were younger (68.79 ± 7.938 vs 74.42 ± 6.751 years, P=0.016) and had smaller waist circumference (87.66 ± 7.835 vs 92.65 ± 7.330 cm, P=0.041), higher level of HDL (1.33 ± 0.363 vs 1.10 ± 0.250 mmol/L, P=0.011) and CTX (0.37 ± 0.185 vs 0.28 ± 0.116 ng/mL, P=0.03) (**Table 1**). In female patients with T2DM, compared to the normal BMD group, the low BMD group were older (69.27 ± 8.093 vs 62.00 ± 10.41 years, P=0.037) and had a lower level of left-hand grip strength (16.63 ± 5.340 vs 19.47 ± 3.663 kg, P=0.038). In female patients without T2DM, compared with the normal BMD group, the low BMD group had a higher level of CTX (0.46 ± 0.195 vs 0.37 ± 0.075 ng/mL, P=0.012) (**Table 2**).

**Table 1:** Baseline clinical and biochemical characteristics of male individuals.

Factors	Non-T2DM (n= 71)			T2DM (n= 96)		
	Normal BMD	Low BMD	P-value	Normal BMD	Low BMD	P-value
	(n= 13)	(n= 58)		(n= 31)	(n= 65)	
A20, pg/mL	76.67 (64.80, 95.10)	82.19 (54.13, 125.72)	0.882	49.47 (39.12, 77.45)	36.53 (26.56, 59.32)	**0.007**
Demographic						
Age, years	74.42 ± 6.751	68.79 ± 7.938	**0.016**	67.42 ± 8.652	67.23 ± 6.830	0.916
Smoking, n(%)	5 (38.46%)	19 (32.76%)	0.751	10 (32.26%)	26 (40.00%)	0.464
Drinking, n(%)	5 (38.46%)	10 (17.24%)	0.13	4 (12.90%)	11 (16.92%)	0.767
Duration of T2DM, years	\	\	\	11.91 ± 7.375	11.67 ± 9.420	0.891
Hypertension, n(%)	7 (53.85%)	32 (55.17%)	0.931	18 (58.06%)	40 (61.54%)	0.745
Use of anti-diabetic drugs						
Insulin, n(%)	\	\	\	15 (48.39%)	23 (35.38%)	0.223
Metformin, n(%)	\	\	\	20 (64.52%)	43 (66.15%)	0.874
Alpha-glucosidase inhibitors, n(%)	\	\	\	7 (22.58%)	15 (23.08%)	0.957
GLP-1 receptor agonists, n(%)	\	\	\	3 (9.68%)	6 (9.23%)	>0.999
DPP-IV inhibitors, n(%)	\	\	\	4 (12.90%)	10 (15.38%)	>0.999
SGLT-2 inhibitors, n(%)	\	\	\	11 (35.48%)	16 (24.62%)	0.268
Use of antihypertensive drugs						
CCBs, n(%)	5 (38.46%)	19 (32.76%)	0.751	13 (41.94%)	17 (26.15%)	0.119
ARBs, n(%)	1 (7.69%)	13 (22.41%)	0.441	5 (16.13%)	14 (21.54%)	0.534
β-blockers, n(%)	2 (15.38%)	1 (1.72%)	0.084	2 (6.45%)	1 (1.54%)	0.243
Anthropometric measurements						
BMI, kg/m^2^	25.18 ± 2.074	24.07 ± 2.836	0.119	25.36 ± 3.138	23.86 ± 2.697	**0.026**
Waist circumference, cm	92.65 ± 7.330	87.66 ± 7.835	**0.041**	93.22 ± 9.737	90.48 ± 7.086	0.251
Hip circumference, cm	97.46 ± 5.060	95.51 ± 4.896	0.222	94.73 ± 5.727	92.55 ± 5.780	0.157
WHR	0.95 ± 0.042	0.92 ± 0.054	0.025	0.98 ± 0.069	0.98 ± 0.052	0.754
Thigh circumference, cm	48.85 ± 3.671	47.61 ± 3.997	0.295	42.93 ± 3.790	40.67 ± 5.087	0.05
Calf circumference, cm	37.14 ± 2.451	35.79 ± 2.485	0.091	35.15 ± 2.659	33.19 ± 3.496	**0.015**
Left-hand grip strength, kg	34.51 ± 5.964	33.33 ± 6.212	0.53	29.79 ± 9.247	27.24 ± 7.501	0.271
Right-hand grip strength, kg	37.10 ± 5.883	35.27 ± 5.275	0.316	32.42 ± 9.360	30.18 ± 9.421	0.368
SPPB score	10.23 ± 1.01	10.48 ± 0.96	0.424	9.73 ± 2.097	9.50 ± 2.973	0.724
Blood biochemical indicators						
Hb, g/L	157.23 ± 12.022	153.67 ± 12.505	0.351	143.81 ± 15.574	141.05 ± 14.155	0.407
ALT, U/L	19.00 (15.00, 23.00)	17.00 (13.00, 22.75)	0.556	17.50 (13.85, 20.30)	16.65 (11.83, 24.08)	0.796
AST, U/L	18.00 (16.00, 21.00)	20.00 (17.00, 23.75)	0.379	18.90 (16.90, 22.00)	17.60 (13.80, 23.53)	0.238
Cr, μmol/L	88.08 ± 9.691	82.66 ± 11.569	0.094	81.23 ± 25.961	79.19 ± 26.394	0.723
FPG, mmol/L	5.40 (4.98, 5.55)	5.00 (4.73, 5.49)	0.337	6.44 (5.11, 8.93)	7.70 (6.00, 11.13)	0.102
HbA1c, %	5.99 ± 0.318	5.80 ± 0.349	0.195	9.84 ± 2.558	9.85 ± 2.198	0.984
FINS, μU/mL	\	\	\	11.50 (7.38, 19.35)	9.42 (5.14, 16.71)	0.343
HOMA-IR	\	\	\	3.34 (1.97, 8.60)	3.47 (1.82, 6.11)	0.77
TC, mmol/L	4.83 ± 1.008	4.94 ± 1.162	0.732	4.89 ± 1.546	4.18 ± 1.192	**0.029**
TG, mmol/L	1.78 (1.06, 2.37)	1.43 (0.98, 2.16)	0.397	1.30 (0.97, 1.65)	1.16 (0.94, 1.73)	0.79
HDL, mmol/L	1.10 ± 0.250	1.33 ± 0.363	**0.011**	1.13 ± 0.279	1.03 ± 0.243	0.104
LDL, mmol/L	2.94 ± 0.807	2.78 ± 0.963	0.548	3.16 ± 1.282	2.66 ± 0.880	0.054
Bone mineral density by DXA						
Lumbar spine 1-4, g/cm^2^	1.12 ± 0.107	0.92 ± 0.123	**<0.001**	1.16 ± 0.250	0.96 ± 0.174	**<0.001**
LS T-Score	0.30 ± 0.982	-1.49 ± 1.138	**<0.001**	0.83 ± 1.226	-1.08 ± 1.237	**<0.001**
Femoral neck, g/cm^2^	0.86 ± 0.060	0.68 ± 0.085	**<0.001**	0.88 ± 0.074	0.69 ± 0.076	**<0.001**
FN T-Score	-0.51 ± 0.384	-1.86 ± 0.622	**<0.001**	-0.36 ± 0.519	-1.83 ± 0.575	**<0.001**
Total hip, g/cm^2^	1.04 ± 0.080	0.87 ± 0.092	**<0.001**	1.06 ± 0.078	0.87 ± 0.106	**<0.001**
TH T-Score	0.10 ± 0.445	-1.08 ± 0.612	**<0.001**	0.14 ± 0.536	-1.12 ± 0.699	**<0.001**
BTMs						
PINP, ng/mL	37.17 ± 11.109	43.51 ± 16.954	0.107	29.70 (20.45, 37.60)	31.95 (24.15, 46.13)	0.361
CTX, ng/mL	0.28 ± 0.116	0.37 ± 0.185	**0.03**	0.27 (0.21, 0.44)	0.31 (0.23, 0.45)	0.324

The serum A20 reference value in males were shown as median (p2.5-p97.5): 60.71 (4.25, 245.13) pg/mL. Values were bolded when the P-value was less than 0.05. T2DM, type 2 diabetes mellitus; BMD, bone mineral density; GLP-1, glucagon-like peptide-1; DPP-IV, dipeptidyl peptidase-4; SGLT-2, sodium-glucose cotransporter-2; CCBs, calcium channel blockers; ARBs, angiotensin II receptor blockers; β-blockers, beta-adrenergic receptor blockers; BMI, body mass index; WHR, waist to hip ratio; SPPB, short physical performance battery; Hb, hemoglobin; ALT, alanine aminotransferase; AST, aspartate aminotransferase; Cr, creatinine; FPG, fasting plasma glucose; HbA1c, glycated hemoglobin; FINS, fasting insulin; HOMA-IR, homeostasis model assessment-insulin resistance; TC, total cholesterol; TG, triglyceride; HDL, high-density lipoprotein; LDL, low-density lipoprotein; DXA, dual-energy x-ray absorptiometry; LS, lumbar spine; FN, femoral neck; TH, total hip; BTMs, bone turnover markers; PINP, procollagen type I intact N-terminal; CTX, c-terminal cross-linking telopeptide of type I collagen.

**Table 2:** Baseline clinical and biochemical characteristics of female individuals.

Factors	Non-T2DM (n= 112)			T2DM (n= 93)		
	Normal BMD	Low BMD	P-value	Normal BMD	Low BMD	P-value
	(n= 10)	(n= 102)		(n= 12)	(n= 81)	
A20, pg/mL	84.84 (70.98, 110.68)	71.48 (53.43, 106.95)	0.298	55.99 (33.14, 78.19)	40.98 (28.14, 60.34)	0.363
Demographic						
Age, years	64.30 ± 5.165	67.36 ± 4.973	0.101	62.00 ± 10.410	69.27 ± 8.093	**0.037**
Smoking, n(%)	0 (0.00%)	0 (0.00%)	>0.999	1 (8.33%)	1 (1.23%)	0.243
Drinking, n(%)	0 (0.00%)	1 (0.98%)	>0.999	0 (0.00%)	0 (0.00%)	>0.999
Duration of T2DM, years	\	\	\	10.84 ± 8.640	13.03 ± 9.358	0.430
Hypertension, n(%)	4 (40.00%)	60 (53.57%)	0.41	4 (33.33%)	36 (44.44%)	0.468
Use of anti-diabetic drugs						
Insulin, n(%)	\	\	\	5 (41.67%)	39 (48.75%)	0.647
Metformin, n(%)	\	\	\	9 (75.00%)	49 (61.25%)	0.524
Alpha-glucosidase inhibitors, n(%)	\	\	\	3 (25.00%)	27 (33.75%)	0.745
GLP-1 receptor agonists, n(%)	\	\	\	1 (8.33%)	12 (15.00%)	>0.999
DPP-IV inhibitors, n(%)	\	\	\	4 (33.33%)	20 (25.00%)	0.504
SGLT-2 inhibitors, n(%)	\	\	\	4 (33.33%)	15 (18.75%)	0.262
Use of antihypertensive drugs						
CCBs, n(%)	3 (30.00%)	37 (36.27%)	>0.999	2 (16.67%)	19 (23.46%)	0.728
ARBs, n(%)	0 (0.00%)	22 (21.57%)	0.206	2 (16.67%)	14 (17.28%)	>0.999
β-blockers, n(%)	1 (10.00%)	7 (6.86%)	0.539	0 (0.00%)	3 (3.70%)	>0.999
Anthropometric measurements						
BMI, kg/m^2^	24.29 ± 2.264	23.34 ± 4.188	0.268	23.47 ± 2.777	24.14 ± 3.431	0.457
Waist circumference, cm	84.90 ± 10.999	82.05 ± 8.315	0.444	87.08 ± 6.772	88.29 ± 8.065	0.597
Hip circumference, cm	97.00 ± 6.377	93.41 ± 5.500	0.116	94.42 ± 6.273	93.04 ± 6.478	0.505
WHR	0.87 ± 0.088	0.88 ± 0.067	0.898	0.92 ± 0.052	0.95 ± 0.067	0.146
Thigh circumference, cm	45.00 ± 3.464	46.44 ± 5.482	0.258	43.08 ± 3.735	42.77 ± 4.212	0.8
Calf circumference, cm	34.90 ± 2.726	34.01 ± 2.703	0.348	33.21 ± 3.401	32.55 ± 2.730	0.539
Left-hand grip strength, kg	19.76 ± 5.398	20.04 ± 5.279	0.88	19.47 ± 3.663	16.63 ± 5.340	**0.038**
Right-hand grip strength, kg	21.91 ± 5.156	21.29 ± 5.117	0.721	20.19 ± 3.909	17.77 ± 5.918	0.095
SPPB score	10.30 ± 1.494	10.00 ± 1.611	0.56	10.17 ± 2.368	9.75 ± 2.505	0.59
Blood biochemical indicators						
Hb, g/L	134.60 ± 6.150	137.13 ± 10.126	0.267	130.83 ± 13.238	126.12 ± 13.149	0.268
ALT, U/L	15.50 (11.50, 24.00)	16.00 (13.00, 21.00)	0.878	15.55 (12.75, 19.80)	16.10 (13.20, 27.35)	0.478
AST, U/L	18.00 (15.00, 23.75)	20.00 (18.00, 23.00)	0.341	17.05 (14.60, 19.15)	18.20 (15.90, 24.50)	0.199
Cr, μmol/L	58.50 (56.50, 68.00)	64.50 (59.00, 68.75)	0.215	51.25 (46.00, 61.10)	56.00 (50.95, 65.30)	0.27
FPG, mmol/L	4.94 (4.56, 5.18)	5.10 (4.87, 5.31)	0.248	7.73 ± 2.663	8.38 ± 3.201	0.451
HbA1c, %	6.03 ± 0.222	5.90 ± 0.261	0.347	9.26 ± 1.417	9.22 ± 1.999	0.927
FINS, μU/mL	\	\	\	13.72 (7.28, 22.47)	12.48 (7.82, 21.12)	0.902
HOMA-IR	\	\	\	4.40 (1.91, 7.96)	4.47 (2.82, 9.18)	0.703
TC, mmol/L	5.79 ± 0.982	5.63 ± 0.924	0.617	5.10 ± 1.492	5.07 ± 1.479	0.951
TG, mmol/L	1.83 ± 0.652	1.78 ± 1.649	0.834	1.62 (1.11, 2.43)	1.63 (1.16, 2.15)	0.894
HDL, mmol/L	1.40 ± 0.258	1.56 ± 0.381	0.111	1.08 ± 0.246	1.24 ± 0.242	0.054
LDL, mmol/L	3.59 ± 0.967	3.23 ± 0.840	0.285	3.22 ± 1.055	3.21 ± 1.207	0.98
Bone mineral density by DXA						
Lumbar spine 1-4, g/cm^2^	0.99 ± 0.057	0.79 ± 0.134	**<0.001**	1.02 ± 0.078	0.82 ± 0.164	**<0.001**
LS T-Score	-0.53 ± 0.521	-2.35 ± 1.223	**<0.001**	-0.28 ± 0.709	-2.00 ± 1.260	**<0.001**
Femoral neck, g/cm^2^	0.78 ± 0.031	0.60 ± 0.083	**<0.001**	0.79 ± 0.066	0.60 ± 0.087	**<0.001**
FN T-Score	-0.59 ± 0.281	-2.21 ± 0.745	**<0.001**	-0.49 ± 0.590	-2.31 ± 0.748	**<0.001**
Total hip, g/cm^2^	0.96 ± 0.071	0.76 ± 0.111	**<0.001**	0.95 ± 0.101	0.75 ± 0.106	**<0.001**
TH T-Score	0.14 ± 0.572	-1.49 ± 0.907	**<0.001**	0.00 ± 0.897	-1.58 ± 0.866	**<0.001**
BTMs						
PINP, ng/mL	52.02 ± 10.516	56.60 ± 18.403	0.246	38.66 ± 9.368	40.39 ± 17.367	0.621
CTX, ng/mL	0.37 ± 0.075	0.46 ± 0.195	**0.012**	0.40 ± 0.219	0.47 ± 0.290	0.332

The serum A20 reference value in females were shown as median (p2.5-p97.5): 61.34 (8.72, 240.92) pg/mL. Values were bolded when the P-value was less than 0.05. T2DM, type 2 diabetes mellitus; BMD, bone mineral density; GLP-1, glucagon-like peptide-1; DPP-IV, dipeptidyl peptidase-4; SGLT-2, sodium-glucose cotransporter-2; CCBs, calcium channel blockers; ARBs, angiotensin II receptor blockers; β-blockers, beta-adrenergic receptor blockers; BMI, body mass index; WHR, waist to hip ratio; SPPB, short physical performance battery; Hb, hemoglobin; ALT, alanine aminotransferase; AST, aspartate aminotransferase; Cr, creatinine; FPG, fasting plasma glucose; HbA1c, glycated hemoglobin; FINS, fasting insulin; HOMA-IR, homeostasis model assessment-insulin resistance; TC, total cholesterol; TG, triglyceride; HDL, high-density lipoprotein; LDL, low-density lipoprotein; DXA, dual-energy x-ray absorptiometry; LS, lumbar spine; FN, femoral neck; TH, total hip; BTMs, bone turnover markers; PINP, procollagen type I intact N-terminal; CTX, c-terminal cross-linking telopeptide of type I collagen.

### 3.2 Association of serum A20 level with anthropometric, SPPB, biochemical and clinical parameters in all subjects

Bivariate correlation analysis was employed to explore the correlation between serum A20 level and other variables. The results showed that serum A20 level was positively associated with drinking (r=0.113, P=0.039), Hb (r=0.133, P=0.015), AST (r=0.149, P=0.006), Cr (r=0.208, P<0.001), HDL (r=0.111, P=0.043) and was negatively associated with T2DM (r=-0.418, P<0.001), waist circumference (r=-0.123,P=0.038), WHR (r=-0.173,P=0.003), FPG (r=-0.396, P<0.001) and HbA1c (r=-0.205, P=0.001). Next, after adjusting for age, sex, smoking, drinking and BMI, the partial correlation analysis revealed that serum A20 level was positively correlated with Hb (r=0.143, P=0.044), AST (r=0.153, P=0.031), Cr (r=0.165, P=0.02) and was negatively associated with occurrence of T2DM (r=-0.352, P<0.001), WHR (r=-0.172, P=0.015), FPG (r=-0.227, P=0.001) and HbA1c (r=-0.205, P=0.004) (**Table 3**).

**Table 3:** Bivariate correlation analysis of variables associated with Serum A20 in all subjects.

Factors	A20 (Ln)			
	r	P-value	Adjusted r	Adjusted P-value
Age	0.043	0.431	\	\
Sex	-0.028	0.604	\	\
Smoking	0.022	0.682	\	\
Drinking	0.113	**0.039**	\	\
T2DM	-0.418	**<0.001**	-0.352	**<0.001**
Hypertension	-0.006	0.919	0.088	0.220
Use of CCBs	-0.021	0.703	0.066	0.357
Use of ARBs	0.038	0.483	0.070	0.329
Use of β-blockers	0.061	0.264	0.033	0.646
BMI	-0.034	0.537	\	\
Waist circumference	-0.123	**0.038**	-0.130	0.068
Hip circumference	0.011	0.856	0.039	0.582
WHR	-0.173	**0.003**	-0.172	**0.015**
Thigh circumference	0.089	0.133	0.092	0.196
Calf circumference	0.072	0.229	0.13	0.068
Left-hand grip strength	-0.023	0.703	0.009	0.905
Right-hand grip strength	-0.018	0.759	-0.004	0.956
SPPB score	-0.059	0.32	-0.139	0.051
Hb	0.133	**0.015**	0.143	**0.044**
ALT	-0.039	0.477	-0.069	0.335
AST	0.149	**0.006**	0.153	**0.031**
Cr	0.208	**<0.001**	0.165	**0.020**
FPG	-0.396	**<0.001**	-0.227	**0.001**
HbA1c	-0.205	**0.001**	-0.205	**0.004**
TC	0.029	0.596	0.018	0.804
TG	0.073	0.182	0.094	0.187
HDL	0.111	**0.043**	0.105	0.139
LDL	-0.061	0.264	-0.109	0.128

Values were bolded when the P-value was less than 0.05. T2DM, type 2 diabetes mellitus; CCBs, calcium channel blockers; ARBs, angiotensin II receptor blockers; β-blockers, beta-adrenergic receptor blockers; BMI, body mass index; WHR, waist to hip ratio; SPPB, short physical performance battery; Hb, hemoglobin; ALT, alanine aminotransferase; AST, aspartate aminotransferase; Cr, creatinine; FPG, fasting plasma glucose; HbA1c, glycated hemoglobin; TC, total cholesterol; TG, triglyceride; HDL, high-density lipoprotein; LDL, low-density lipoprotein.

### 3.3 Association of serum A20 level with BMD and BTMs

The results of Pearson analysis showed that serum A20 level was significantly associated with BMD (r=0.253, P=0.032) and corresponding T-score (r=0.255, P=0.031) of lumbar spine 1-4 in male patients with T2DM. However, no correlation was shown between serum A20 level with BMD of femoral neck, BMD of total hip and BTMs. Besides, no correlation was shown between serum A20 level with BMD and BTMs in other groups (**Table 4**).

**Table 4:** Linear correlation analysis of A20 with BMD, T-Scores and BTMs.

Factors	Males				Postmenopausal females			
	Non-T2DM		T2DM		Non-T2DM		T2DM	
	r	P-value	r	P-value	r	P-value	r	P-value
LS 1-4 BMD	0.12	0.318	0.253	**0.032**	0.08	0.403	0.048	0.679
LS 1-4 T-Score	0.128	0.286	0.255	**0.031**	0.08	0.403	0.126	0.28
FN BMD	-0.12	0.318	0.103	0.380	-0.006	0.947	0.085	0.463
FN T-Score	-0.12	0.319	0.174	0.134	0.006	0.951	0.077	0.504
TH BMD	-0.147	0.22	0.069	0.556	0.047	0.624	0.170	0.142
TH T-Score	-0.143	0.234	0.142	0.224	0.045	0.634	0.174	0.132
PINP	0.036	0.766	-0.110	0.351	-0.027	0.774	0.190	0.100
CTX	0.091	0.451	0.076	0.459	-0.089	0.352	-0.120	0.309

Values were bolded when the P-value was less than 0.05. BMD, bone mineral density; BTMs, bone turnover markers; T2DM, type 2 diabetes mellitus; LS, lumbar spine; FN, femoral neck; TH, total hip; PINP, procollagen type I intact N-terminal; CTX, c-terminal cross-linking telopeptide of type I collagen.

### 3.4 Multivariate regression analysis of variables contributing to lumbar spine 1-4 BMD in male patients with T2DM

Firstly, univariate correlation analysis was employed to figure out the potential confounders of BMD. The results showed that the serum A20 level, duration of T2DM, hypertension, use of insulin and GLP-1 receptor agonists were positively correlated with lumbar spine 1-4 BMD and FPG was negatively correlated with lumbar spine 1-4 BMD (P<0.1). Next, multivariate regression analysis showed that serum A20 level (Beta=0.047; 95% CI: 0.007-0.086; P=0.024) and use of insulin (Beta=0.084; 95% CI: 0.002-0.166; P=0.049) were positively correlated with lumbar spine 1-4 BMD and FPG was negatively correlated with lumbar spine 1-4 BMD (Beta=-0.011; 95% CI: -0.021-0.000; P=0.047) after adjusting for confounding factors (**Table 5**).

**Table 5:** Multivariate regression analysis of variables contributing to lumbar spine 1-4 BMD in male patients with T2DM.

Factors	Univariate analysis			Multivariate analysis		
	Beta	95% CI	P-value	Beta	95% CI	P-value
A20 (Ln)	0.045	0.005, 0.086	**0.032**	0.047	0.007, 0.086	**0.024**
Age	0.002	-0.003, 0.007	0.362			
Smoking						
no	\	\	\			
yes	-0.001	-0.071, 0.070	0.983			
Drinking						
no	\	\	\			
yes	0.006	-0.087, 0.098	0.903			
Duration of T2DM	0.004	0, 0.008	0.087	0.002	-0.007, 0.003	0.522
Hypertension						
no	\	\	\			
yes	0.062	-0.007,0.131	0.083	0.055	-0.023,0.133	0.174
Use of insulin						
no	\	\	\			
yes	0.101	0.034, 0.168	**0.004**	0.084	0.002, 0.166	**0.049**
Use of metformin						
no	\	\	\			
yes	-0.003	-0.074, 0.068	0.939			
Use of alpha-glucosidase inhibitors						
no	\	\	\			
yes	-0.021	-0.102, 0.059	0.603			
Use of GLP-1 receptor agonists						
no	\	\	\			
yes	0.150	0.032, 0.267	**0.014**	0.174	-0.071, 0.305	0.226
Use of DPP-IV inhibitors						
no	\	\	\			
yes	-0.008	-0.106, 0.090	0.872			
Use of SGLT-2 inhibitors						
no	\	\	\			
yes	0.028	-0.048, 0.104	0.468			
Use of CCBs						
no	\	\	\			
yes	0.027	-0.047,0.101	0.474			
Use of ARBs						
no	\	\	\			
yes	0.037	-0.047,0.121	0.389			
Use of β-blockers						
no	\	\	\			
yes	0.051	-0.184,0.296	0.671			
BMI	0.005	-0.006, 0.017	0.372			
Waist circumference	0.003	-0.003, 0.008	0.366			
Hip circumference	0.002	-0.006, 0.010	0.597			
WHR	0.313	-0.483, 1.108	0.444			
Thigh circumference	0.005	-0.005, 0.014	0.353			
Calf circumference	0.003	-0.010, 0.017	0.623			
Left-hand grip strength	-0.001	-0.007, 0.005	0.742			
Right-hand grip strength	-0.001	-0.005, 0.004	0.807			
SPPB score	-0.008	-0.025, 0.009	0.334			
Hb	0	-0.002, 0.003	0.739			
ALT	-0.001	-0.004, 0.002	0.414			
AST	0.001	-0.004, 0.006	0.761			
Cr	0.001	0, 0.002	0.136			
FPG	-0.009	-0.019, 0.001	0.078	-0.011	-0.021, 0	**0.047**
HbA1c	0.001	-0.014, 0.016	0.89			
FINS	0	-0.001, 0.001	0.512			
HOMA-IR	0	-0.004, 0.004	0.963			
TC	-0.003	-0.029, 0.022	0.794			
TG	-0.01	-0.050, 0.030	0.628			
HDL	-0.024	-0.160, 0.111	0.726			
LDL	-0.004	-0.040, 0.032	0.828			
PINP	-0.001	-0.003, 0.001	0.232			
CTX	-0.078	-0.247, 0.090	0.364			

Values were bolded when the P-value was less than 0.05. T2DM, type 2 diabetes mellitus; BMD, bone mineral density; CI, confidence interval; GLP-1, glucagon-like peptide-1; DPP-IV, dipeptidyl peptidase-4; SGLT-2, sodium-glucose cotransporter-2; CCBs, calcium channel blockers; ARBs, angiotensin II receptor blockers; β-blockers, beta-adrenergic receptor blockers; BMI, body mass index; WHR, waist to hip ratio; SPPB, short physical performance battery; Hb, hemoglobin; ALT, alanine aminotransferase; AST, aspartate aminotransferase; Cr, creatinine; FPG, fasting plasma glucose; HbA1c, glycated hemoglobin; FINS, fasting insulin; HOMA-IR, homeostasis model assessment for insulin resistance; TC, total cholesterol; TG, triglyceride; HDL, high-density lipoprotein; LDL, low-density lipoprotein; PINP, procollagen type I intact N-terminal; CTX, c-terminal cross-linking telopeptide of type I collagen.

### 3.5 Multivariable-adjusted OR for the correlation of serum A20 level with increased presence of low BMD in male patients with T2DM

**Figure 3:**
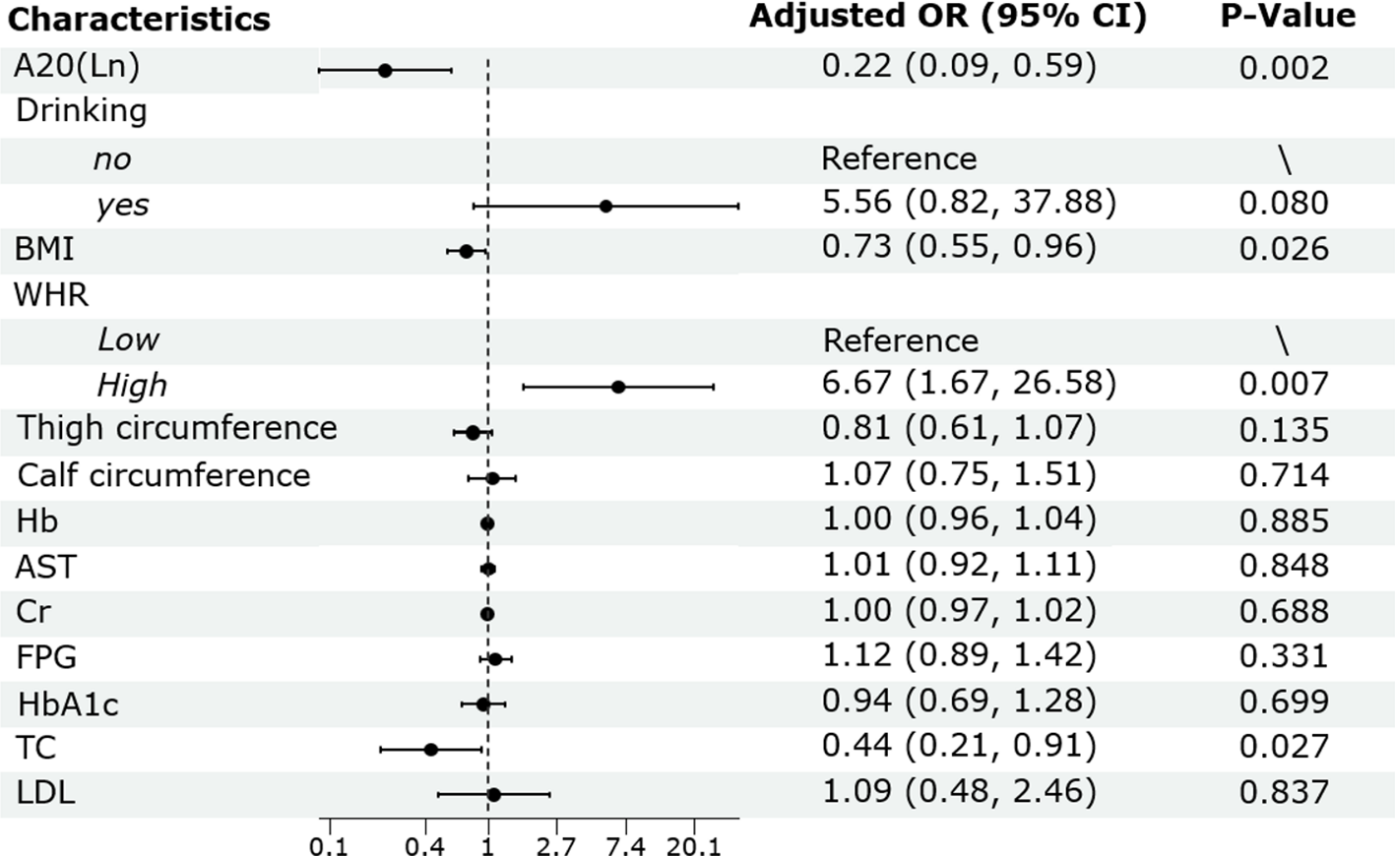
Binary logistic regression analysis of the risk factors of low BMD in male patients with T2DM. BMD, bone mineral density; T2DM, type 2 diabetes mellitus; OR, odds ratio; CI, confidence interval; BMI, body mass index; WHR, waist to hip ratio; Hb, hemoglobin; AST, aspartate aminotransferase; Cr, creatinine; FPG, fasting plasma glucose; HbA1c, glycated hemoglobin; TC, total cholesterol; LDL, low-density lipoprotein.

Binary logistic regression analysis was conducted to assess the independent association between serum A20 level and low BMD. The result showed that after adjusting for drinking, BMI, WHR, thigh circumference, calf circumference, Hb, AST, Cr, FPG, HbA1c, TC and LDL, serum A20 level remained as a negative predictor for low BMD (OR:0.22; 95% CI: 0.09-0.59; P =0.002). Besides, BMI (OR: 0.73; 95% CI: 0.55-0.96; P =0.026) and TC (OR: 0.44; 95% CI: 0.21-0.91; P =0.027) were negative predictors for low BMD and higher WHR was a positive predictor for low BMD (OR: 6.67; 95% CI: 1.67-26.58; P =0.007) (**Figure 3**).

## 4 Discussion

Glucose metabolism and A20 could regulate each other reciprocally. One the one hand, previous studies suggested that high glucose environment or cytokines like TNF-α can initially stimulate the early production of A20 mRNA in various cell types (25–27). However, sustained hyperglycemia, may cause A20 mRNA to undergo N^6^-methyladenosine(m^6^A) modification, reducing its stability, accelerating its degradation and ultimately lowering A20 expression (28). At the protein level, studies have revealed significant reductions in A20 under high glucose conditions (29). For instance, A20 in smooth muscle cells (SMCs) may undergo ubiquitination and degradation due to glycosylation modifications when exposed to high glucose (30). Consistently, a study reported that patients with T2DM and LADA had significantly lower mRNA and protein expression level of A20 in peripheral blood mononuclear cells compared to the control group (31). On the other hand, the decline or pathological deficiency of A20 level may profoundly impact the blood glucose concentration and insulin production, particularly by modulating inflammatory signaling pathways and β-cell functions. Overexpressed A20 in islet could block NF-κB activation through reducing the induction of pro-inflammatory cytokines (32). Besides, A20 maintained β-cell integrity and survival by regulating apoptotic signaling (33). In addition to its role in controlling NF-κB signaling, A20 could down-regulate c-Jun N-terminal kinase (JNK) pathway and up-regulate v-Akt murine thymoma viral oncogene homolog (AKT)-dependent survival pathway in β-cell, thus protected β-cells from intrinsic apoptotsis (34). Lowered A20 level in human islet cells led to impaired insulin secretion induced by glucose, diminished expression of β-cell regulatory genes and transcription factors which are essential for the β-cell maturation and normal function (20).

Previous studies showed that patients with osteoporosis had lower level of Hb compared to the control group (35). Hemoglobin plays an antioxidant role in the preservation of bone health by safeguarding against oxidative stress-induced bone resorption and promoting bone formation (36). Higher WHR indicates more visceral white adipose tissue (WAT). Increased WAT is linked to systemic inflammation (37), oxidative stress, insulin resistance and endothelial dysfunction (38). In addition to IL-6, TNF-α, which can be partially secreted by WAT, WAT could also regulate bone homeostasis by secreting a variety of adipokines, such as leptin, adiponectin and resistin, etc. (39). For example, resistin could directly increase the circulating levels of IL-6 and TNF-α through NF-κB pathway and thus affect osteoclast differentiation (40). The multivariate logistic regression analysis found that higher WHR increased the risk of bone loss, which was consistent with previous studies (41).

A20 could influence bone homeostasis by participating in the regulation of bone formation and resorption processes. A20 non-specific knockout mice exhibited thinner trabeculae compared to wild-type mice (42). A20 deficiency stimulates receptor activator of nuclear factor kappa-B (RANK)-dependent osteoclasts formation and activity by persistently activating the NF-kB pathway (22, 43), upregulating osteoclastic markers such cathepsin K and matrix metallopeptidase (MMP-9) (44) and increasing calcium and phosphate absorption (45). Animal experiments corroborate that specific knockout of A20 in osteoclasts considerably decreased trabecular volume and density without noticeable effects on cortical bone (22). Current research explores A20 as a potential therapeutic target to inhibit osteoclast activity (46). Additionally, A20 influences bone formation by affecting various types of cells. A20 could protect osteoblasts from TNF-α-induced apoptosis and promote osteogenic differentiation (26). Though little understood, the influence of A20 on MSCs suggest its role in enhancing alkaline phosphatase activity and Ca^2+^ mineralized nodule accumulation during osteogenic induction mediated by γ-aminobutyric acid (GABA) (47). A20 has been shown to mitigate NF-κB activation in response to high glucose, reducing endothelial damage and apoptosis (27). Based on this, it can be speculated that A20 may influence osteogenesis by affecting the vascular endothelium closely coupled with osteogenesis, such as the H-type vessels in the metaphysis of long bones. However, this hypothesis requires further research to be confirmed.

A20 was found to be positively associated with lumbar spine 1-4 BMD rather than femoral neck or total hip BMD. The lumbar spine contains a higher proportion of trabecular bone compared to cortical bone (48). Chronic inflammation affects trabecular and cortical bone to different extent, typically causing more bone loss in trabecular regions (49). Therefore, the lumbar spine may be more responsive to A20’s regulation of inflammation.

The positive correlation between A20 and bone mass was shown in males but not females. Recent studies found that in both early and late postmenopausal females, bone mass loss remained at a high rate (50, 51). Although the peak bone loss in females occurs between the ages of 50 and 55, studies have shown that in individuals aged 67–69 years, bone loss in females is 2–5 times greater than in males (52). In females, the impact of estrogen deficiency persists throughout both the early and late stages of postmenopause (53). The abrupt decline in estrogen level triggers rapid bone loss over months to years after menopause, then followed by a second peak around the age of 70, possibly driven by secondary hyperparathyroidism resulting from early-phase bone loss (54, 55). In males, the gradual decline in sex hormones leads to a slow and sustained loss of bone mass (56, 57). Although increasing studies suggests that estrogen may play a more significant role than androgens in male osteoporosis (58–60), estrogen levels in older adults males remain higher than those in postmenopausal females (61, 62). Accelerated bone loss associated with estrogen withdrawal is mediated in part by increased oxidative stress and inflammation. The estrogen decline induces T cell activation, which elevates inflammatory cytokines like TNF-α and IL-1 in the bone marrow and circulation (63–66). TNF-α promotes RANKL production, enhancing osteoclast differentiation and suppresses osteogenic gene expression (67–69). Consequently, the significantly lower levels of estrogen in postmenopausal females may lead to more severe inflammation-induced bone loss than males, potentially overshadowing the regulatory role of A20 in inflammation.

This study provides us bountiful clues about the association between serum A20 level with bone metabolism, however, this study had several limitations. Firstly, cross-sectional design in this study was unable to determine causal relationships and perspective trials are necessary to confirm these results. Secondly, clinical trials with larger samples are necessary to confirm the different results between gender. Lastly, basic experimental researches will provide novel insights about how A20 regulates diabetic osteopathy.

## Data Availability

The original contributions presented in the study are included in the article/supplementary material. Further inquiries can be directed to the corresponding author.
